# Nitrochalcones: Pharmacological Activities and Therapeutic Potential

**DOI:** 10.3390/ijms27062711

**Published:** 2026-03-16

**Authors:** Erika Madeleyne Ramos-Rivera, Nancy Romero-Ceronio, Oswaldo Hernández-Abreu, Cuauhtémoc Alvarado-Sánchez, Quirino Torres-Sauret, Manuel Velasco-Ximello, Heidi Beatriz Montejo-Méndez, Jorge Cortez-Elizalde, Nadia Landero-Valenzuela, Luis Fernando Roa de la Fuente, Rosalía Torralba Sánchez, Lucero Vázquez-Cruz, Miguel Ángel Vilchis-Reyes, Alam Yair Hidalgo

**Affiliations:** 1 División Académica de Ciencias Básicas, Universidad Juárez Autónoma de Tabasco, Carretera Cunduacán-Jalpa Km 1, Colonia La Esperanza, Cunduacán 86690, Tabasco, Mexico; erika.ramos@ujat.mx (E.M.R.-R.); nancy.romero@ujat.mx (N.R.-C.); oswaldohernandezabreu@gmail.com (O.H.-A.); calvarados09@gmail.com (C.A.-S.); quirino.sauret@gmail.com (Q.T.-S.); jorge.cortez@ujat.mx (J.C.-E.); fernando.roa@ujat.mx (L.F.R.d.l.F.); 2 Complejo Regional Mixteca, Campus Izúcar de Matamoros, Benemérita Universidad Autónoma de Puebla, Carretera Atlixco-Izúcar de Matamoros 141, San Martín Alchichica, Izúcar de Matamoros 74570, Puebla, Mexico; manuel.velascoxim@correo.buap.mx (M.V.-X.); rosalia.torralbasan@correo.buap.mx (R.T.S.); 3 División Académica de Ciencias Biológicas, Universidad Juárez Autónoma de Tabasco, Carretera Villahermosa-Cárdenas Km. 0.5 S/N Entronque a Bosques de Saloya, Villahermosa 86150, Tabasco, Mexico; heidibmm@gmail.com (H.B.M.-M.); lucerovc@gmail.com (L.V.-C.); 4 Departamento de Horticultura, Universidad Autónoma Agraria Antonio Narro, Calzada Antonio Narro 1923, Buenavista, Saltillo 25315, Coahuila, Mexico; nadialandero00@gmail.com; 5 Tecnológico Nacional de México—Instituto Tecnológico Superior de Comalcalco, Carretera Vecinal, Comalcalco-Paraíso Km 2, R/a Occidente 3ra Sección, Comalcalco 86650, Tabasco, Mexico

**Keywords:** chalcones, nitrochalcones, pharmacology, multitarget

## Abstract

Chalcones are compounds containing an α,β-unsaturated carbonyl group that have been studied due to their structural simplicity, ease of synthesis, and broad spectrum of biological activities. Within this family, nitrochalcones have gained relevance due to the influence of the nitro group (–NO_2_) on the modulation of their electronic properties, chemical reactivity, and pharmacological behavior. This review presents a critical analysis of advances in the biological activities and possible mechanisms of action of nitrochalcones and their derivatives, with an emphasis on their structure–activity relationships and therapeutic potential. The available evidence shows that nitrochalcones and their derivatives act as multitarget molecules, capable of intervening in key biological processes such as oxidative stress, mitochondrial dysfunction, inflammation, and cell signaling pathways associated with proliferation and apoptosis. Several studies report anticancer, anti-inflammatory, antiparasitic, antimicrobial, antifungal, and cardiovascular activities, often with favorable selectivity toward pathological cells over healthy cells.

## 1. Introduction

Nitrochalcones are a notable subclass of chalcones, characterized by the presence of one or more nitro (–NO_2_) groups in one or both aromatic rings of the α,β-unsaturated enone system [1]. Interest in nitrochalcones is reinforced by the widespread historical use of nitroaromatic compounds in therapeutics. For more than 70 years, these compounds have been utilized as anti-infective agents, demonstrating efficacy against anaerobic bacteria. Nifurtimox and benznidazole, nitroheterocyclic derivatives, have been the standard treatment for Chagas disease since the 1960s, emphasizing the pharmacological importance of the nitro group [2].

From a biological standpoint, the core structure (defined as the chalcone lacking substituents) of these compounds, particularly the α,β-unsaturated system, manifests a multitude of pharmacological properties [3]. Consequently, nitrochalcones capitalize on the acknowledged biological potential of the chalcone framework, coupled with the pronounced electroattractive effect of the nitro group, yielding a notable array of pharmacological activities [4]. Furthermore, the α,β-unsaturated double bond conjugated to the carbonyl group is hypothesized to be a pivotal part of the pharmacophore, and its modification or elimination typically results in a substantial loss of activity. These molecules can also exist as cis and trans geometric isomers, with the trans isomer being the most stable and most frequently associated with relevant biological effects [5].

From a physicochemical perspective, the nitro group (–NO_2_) is characterized by its strong electron-withdrawing nature, arising from the positive charge on the nitrogen atom and its ability to withdraw electron density from the aromatic system through inductive and resonance effects. In aromatic rings, this behavior leads to electronic deactivation and significant changes in molecular polarity. These factors can favor the interaction of nitrochalcones with nucleophilic sites in proteins, such as enzymes. This, in turn, can promote biological inhibition [6,7].

The reactivity of the nitro group has been widely utilized in medicinal chemistry, particularly in the design of functional compounds and self-sacrificing systems. In these applications, the nitro group can function as a crucial activator, linker, or reporter unit [8].

The synthetic accessibility of nitrochalcones has facilitated their systematic study, thus enabling the generation of structurally diverse libraries. This has promoted their use both as direct bioactive molecules and as key intermediates in the synthesis of heterocyclic compounds of pharmaceutical interest [9].

Several reviews have shown that both natural and synthetic chalcones are privileged scaffolds in medicinal chemistry and drug discovery, with a diverse range of biological activities [10,11,12,13]. Recently, chalcones from natural sources have been reviewed, covering multiple in vitro and in vivo biological activities, including structure-activity relationship studies and mechanisms of action, and these studies suggest that clinical and preclinical research is limited [14,15]. The multiple pharmacological activities of natural chalcones and their simple chemical structure inspired the synthesis of numerous chalcone derivatives [16]. Today, numerous reviews focus on the synthesis and diverse biological activities of chalcone derivatives. Some of these reviews covered several biological activities, SAR, mechanism of action, preclinical and clinical evidence, and molecular docking studies [17,18,19,20,21]. As is well known, the biological activity of chalcones is determined by the functional groups on the A- and B-rings. In this sense, generating functionalized chalcone derivatives is straightforward from simple aromatic compounds. For this reason, several investigations have been conducted with particular subgroups of chalcones. However, reviews concerning methods of synthesis and their pharmacological application have been minimally studied [22]. For example, Kubiak et al. conducted a review that discusses the synthesis of fluorinated chalcones and their multiple biological activities, particularly their antibacterial, antiviral, and anticancer properties (Table 1) [23]. With this in mind, and considering the increase in the number of publications related to a subgroup of chalcones (nitrochalcones).

**Figure 1:**
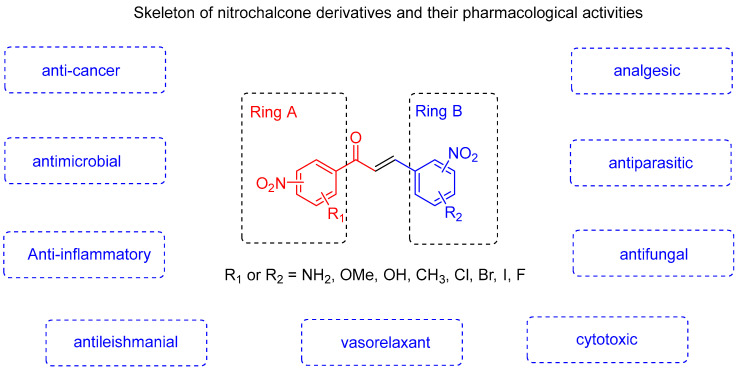
Pharmacological activities of nitrochalcones.

The objective of this review is to analyze and systematize advances in research on nitrochalcones and their derivatives, covering multiple aspects of their pharmacological activities (Figure 1). To the best of our knowledge, there is currently no integrated review of nitrated chalcones available. In light of the considerable heterogeneity in the pharmacological effects documented in the extant literature, it is imperative to organize these findings systematically to underscore their pertinence and therapeutic potential.

Likewise, this work study is to integrate the extant evidence on structure-activity relationships and the impact of the nitro group on the biological behavior of these molecules. This review also aims to identify trends, limitations, and opportunities for future research, providing a basis for further work on the rational design of new derivatives and the development of therapeutic strategies based on nitrochalcones.

## 2. Research Design and Search Strategies

To write this paper, a literature search was conducted to identify experimental pharmacological studies on chalcones containing at least one nitro group (nitrochalcones). The search was conducted in the main international scientific databases: Scopus, Web of Science (WoS), PubMed, ScienceDirect, and Google Scholar. The time frame spanned from 2000 to August 2025, with particular emphasis on the last decade, given the substantial surge in publications on nitrochalcones and their derivatives.

Combinations of keywords in English were used with Boolean operators (AND, OR), including:

- “nitrochalcone” OR “nitro chalcone.”
- “nitrochalcone” AND “anticancer.”
- “nitrochalcone” AND “anti-inflammatory.”
- “nitrochalcone” AND “antimicrobial.”
- “nitrochalcone” AND “antiparasitic.”
- “chalcone derivatives” AND “nitro group.”
- “structure–activity relationship” AND “nitrochalcone.”

A total of 38 studies were included in the review, all of which address the pharmacological evaluation of nitrochalcones. The information was methodically arranged into thematic categories to facilitate the identification of trends, convergences, and knowledge gaps. This methodological approach enabled the resumption of the analysis of available results and the proposal of an integrative interpretation of the role of nitrochalcones in medicinal chemistry.

### 2.1. Study Eligibility Criteria

- Inclusion Criteria
  1. Structurally, chalcones must have at least one nitro group.
  2. That nitrochalcones have significant biological effects.
  3. Articles reported in English, except for the first report on nitrochalcones.
  4. Original articles, reviews, short communications, and mechanistic studies.
- Exclusion Criteria
  1. Studies that only provide a summary of nitrochalcones but no evaluation of pharmacological activity.
  2. Studies that had no pharmacological relevance.
  3. Studies with incomplete or irreproducible experimental data.
  4. Articles not directly related to chalcones or nitro derivatives.

### 2.2. Data Organization and Justification of the Narrative Approach

Relevant information was systematically extracted, including:

- Position of the nitro group (ortho, meta, para) in rings A and/or B.
- Type of additional substituents.
- Reported biological activity (IC_50_, EC_50_, MIC, etc.).
- Biological model used.
- Associated mechanistic proposals (Michael-type interactions, ROS generation, enzyme inhibition).

Given the marked heterogeneity of the studies identified in the literature search, a qualitative synthesis was chosen over a quantitative meta-analysis. The studies included showed significant variability in terms of experimental designs, biological models used (in vitro, in vivo, and mechanistic studies), types of biochemical assays, and pharmacological parameters reported (IC_50_, EC_50_, MIC, selectivity indices, ROS levels, and enzyme markers), as well as substantial structural differences in the nitrochalcone skeletons evaluated.

Likewise, methodological inconsistencies were observed in relation to experimental conditions, concentrations evaluated, incubation times, and cell systems used, which prevent the statistical standardization necessary for robust quantitative integration. The absence of comparable, uniformly reported data limits the applicability of statistical tools used in formal meta-analysis.

Consequently, a structured narrative review approach was adopted, allowing for the critical integration of available evidence, the identification of trends in the structure–activity relationship (SAR), the analysis of mechanistic patterns associated with the nitro group, and the discussion of pharmacological implications from a molecular perspective. This approach favors a contextualized interpretation of the findings and is particularly appropriate in emerging or developing fields, where methodological diversity still prevents the consolidation of homogeneous data for quantitative analysis.

## 3. Nitrochalcones as Privileged Multitarget Scaffolds

The concept of privileged scaffolds refers to chemical structures that interact with multiple biological targets and elicit a wide range of pharmacological activities [24]. Nitrochalcones satisfy this criterion due to the presence of an α,β-unsaturated system and nitro group, which enables participation in a variety of molecular interactions and modulation of multiple biological pathways. Several studies have demonstrated that nitrochalcones can interact with a variety of biological targets, including enzymes, receptors, regulatory proteins, and pivotal cellular components.

### 3.1. Anticancer Activity

**Figure 2:**
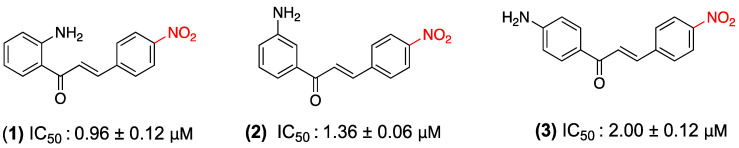
Chalcones **1**–**3** were evaluated in HT-29, LS180, LoVo, and LoVo/DX cells.

The anticancer activity of nitrochalcones is among the most studied pharmacological properties. In 2019, Kozlowska et al. investigated this effect in three nitrochalcone derivatives substituted at position 4 of ring B (**1**–**3**) in human colon cancer cell lines (HT-29, LS180, LoVo, and LoVo/DX). Utilizing the SRB assay, it was determined that these compounds manifest significant cytotoxic activity. Of the three isomers, 2′-amino-4-nitrochalcone (**1**) was the most effective, with significantly lower IC_50_ values (0.96–2.24 μg/mL) than cisplatin (16.73 μg/mL in HT-29). Despite the absence of a comprehensive understanding of the underlying mechanism, a hypothesis has been postulated. The hypothesis suggests that the nitro group at position 4 functions as an electron acceptor, thereby inducing selective apoptosis in cancer cells rather than necrosis. This characteristic, in conjunction with its superior activity against resistant lines such as LoVo/DX, is regarded as a significant advantage. These findings position the nitrochalcones as potential therapeutic candidates for the development of new, more effective, and selective anticancer agents (Figure 2) [25].

**Figure 3:**
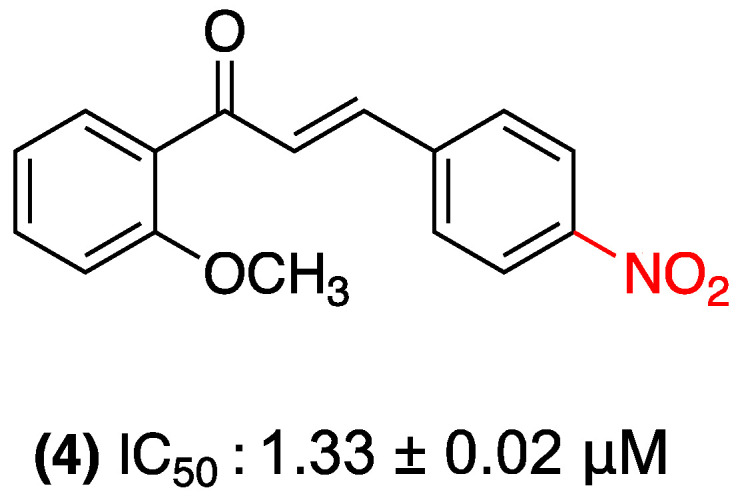
Nitrochalcone **4** was evaluated in MCF-7SC cells.

In 2022, Ahn and colleagues identified 2′-methoxy-4-nitrochalcone (**4**) as a promising compound among 17 polymethoxylated chalcones evaluated, demonstrating potent anticancer activity against breast cancer stem cells (MCF-7SC; IC_50_ = 1.33 μM). The derivative induced a dose-dependent apoptosis by up-regulating key markers (caspase-7, c-PARP, Bax/Bcl-2). Furthermore, the results indicated a significant inhibition of cell migration, suggesting a potential antimetastatic effect. Mechanistically, (**4**) suppressed epithelial–mesenchymal transition (by decreasing Snail/Vimentin and increasing E-cadherin) and reduced the population of cancer stem cells (CD44+/CD24−), which are critical targets for therapy resistance. In addition, the study observed a decline in stemness markers (CD44, MRP1, β-catenin) and a significant inhibition of GSK3β activity (89% at 10 μM). These results were corroborated by molecular docking studies, which revealed a high binding affinity (−7.5 to −6.8 kcal/mol) (Figure 3). Predicted pharmacokinetic analyses indicate that (**4**) meets Lipinski’s criteria and has a favorable absorption and oral bioavailability profile. These findings suggest that this nitrochalcone is the most promising compound in the study [26].

**Figure 4:**
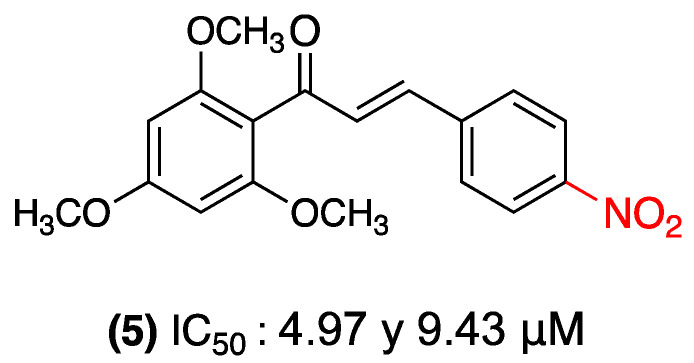
Nitrochalcone **5** was evaluated in ESCC.

In a similar vein, a 2022 study reported that nitrochalcones demonstrated noteworthy antitumor activity against esophageal squamous cell carcinoma (ESCC). Yan Yang and colleagues synthesized and evaluated 20 chalcone derivatives, identifying nitrochalcone (**5**) as the most active. This compound exhibited a dose- and time-dependent antiproliferative effect in ESCC cell lines (KYSE-450 and Eca-109), with IC_50_ values of 4.97 µM and 9.43 µM, respectively. The mechanism of action of (**5**) involves the generation of reactive oxygen species (ROS), which trigger cell-cycle arrest in the G2/M phase and apoptosis. These effects were corroborated by increases in proapoptotic proteins. In an in vivo study employing a mouse xenograft model, the compound (**5**) significantly reduced tumor volume without affecting body weight, indicating a favorable toxicity profile (Figure 4). The results of this study demonstrate the potential of nitrochalcones as therapeutic candidates for ESCC. The findings suggest that this compound may function by inducing oxidative stress and promoting apoptosis [27].

**Figure 5:**
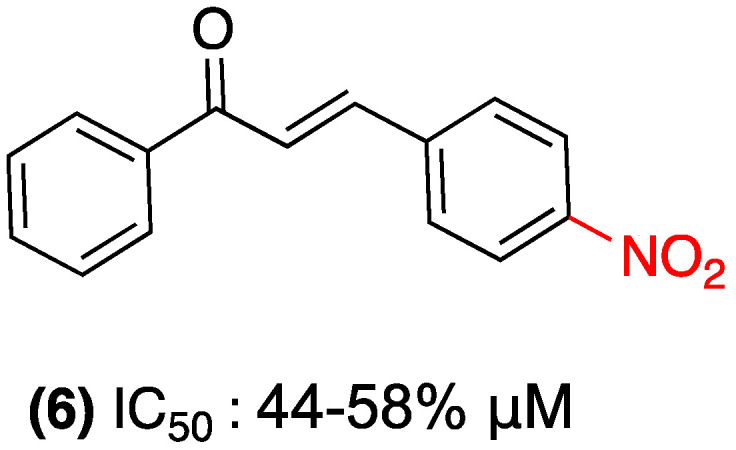
Nitrochalcone **6** was evaluated in MCF-7 and MDA-MB-231.

In 2023, Martins et al. investigated 4-nitrochalcone (**6**), which demonstrated selective antitumor activity against breast cancer in an in vivo Ehrlich carcinoma model, resulting in a 44–58% reduction in tumor volume. The compound exhibited preferential toxicity in the MCF-7 (SI = 3.68) and MDA-MB-231 cell lines, with minimal damage to non-tumor cells (HB4a). Its mechanism of action involves dysfunctional autophagy (increased LC3-II without p62 degradation) and mTORC1 inhibition, which reduces SGK1 phosphorylation and alters tumor energy metabolism (decreased lactate/pyruvate ratio). The molecule demonstrated no substantial toxicity (25 mg/kg, oral), adhered to Lipinski’s rules, and did not result in hematological or organ damage (Figure 5) [28].

**Figure 6:**
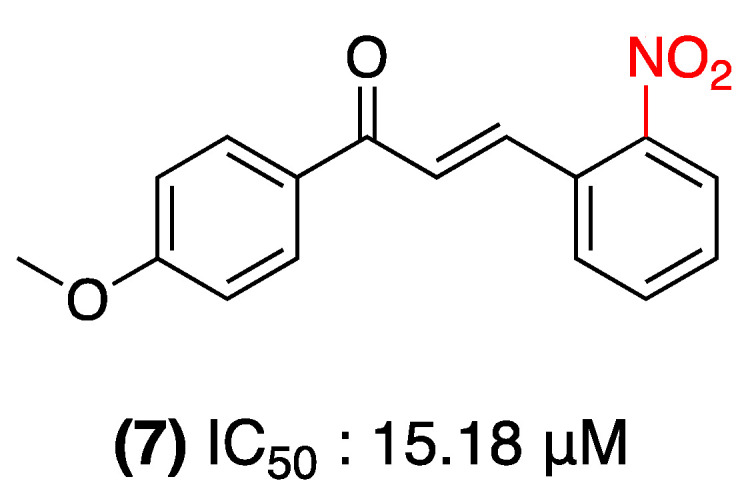
Nitrochalcone **7** was evaluated in HCT116 cells.

In the study conducted by Machado et al. in 2023, 4′-Methoxy-2-nitrochalcone (**7**) was evaluated as a potent inhibitor of the anti-apoptotic protein MCL-1, which plays a pivotal role in tumor resistance. This nitrochalcone derivative exhibited a significant inhibitory effect on HCT116 cell proliferation, with an IC_50_ value of 15.18 μM after 24 h of treatment. The compound induced various biological effects, including plasma membrane rupture at 15.58 μM and apoptosis at 7.79 μM. Molecular docking studies revealed the binding of the compound to the active site of MCL-1 via π-π interactions with Phe270 and hydrogen bonds with Leu267/Thr266 (Figure 6) [29].

To systematize the available evidence and facilitate comparison between studies, Table 2 presents a structured summary of the nitrochalcones evaluated for anticancer activity, including their substitution pattern, proposed molecular targets or pathways, biological model used, reported potency, selectivity, and main experimental limitations. The comparative organization of these data allows us to identify clear trends in the structure-activity relationship, particularly regarding the position of the nitro group and its impact on oxidative stress induction, apoptosis, and modulation of cell signaling pathways. Likewise, the inclusion of the methodological limitations reported in the original studies contributes to a critical interpretation of the evidence and helps prevent overestimating biological efficacy.

### 3.2. Antimicrobial Activity

**Figure 7:**
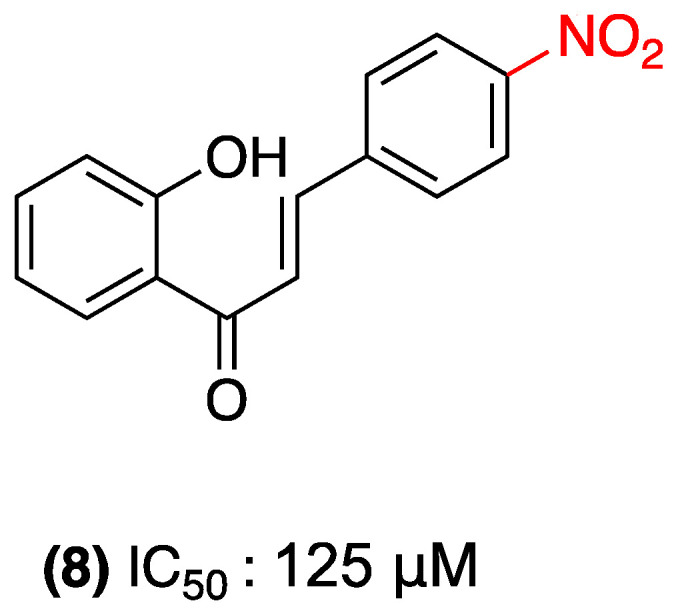
Nitrochalcone **8** evaluated in HCT116 cells.

A number of studies have examined the antimicrobial activity of nitrochalcones, yielding promising results. For instance, in 2014, Ibrahim’s group evaluated the antimicrobial and antifungal activity of a series of chalcones, including 2′-hydroxy-4-nitrochalcone (**8**). These compounds demonstrated moderate activity against the Gram-positive bacteria *Enterococcus faecalis* (14 ± 0.53 mm) and *Bacillus cereus* (12 ± 0.55 mm), with an MIC of 125 µg/mL (Figure 7). However, when tested against the Gram-negative bacteria *Klebsiella pneumoniae* (7 ± 0.40 mm) and *Pseudomonas aeruginosa* (8 ± 0.22 mm), the minimum inhibitory concentration (MIC) increased to 500 µg/mL. In addition to the bacterial assay, this compound was evaluated as an antifungal agent against *Aspergillus fumigatus* (7 ± 0.43 mm) and *Candida glabrata* (6 ± 0.45 mm) at an MIC of 250 µg/mL, indicating low effectiveness relative to the standard control [30].

**Figure 8:**
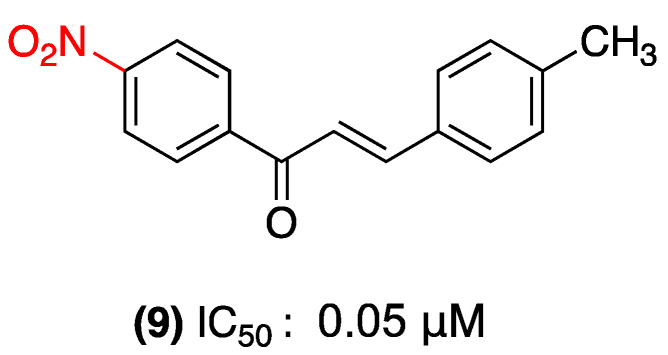
Nitrochalcone **9** was evaluated in *S. aureus* ATCC 25923, *S. aureus* MRSA ATCC 33591, and *C. albicans* MYA 2876.

Almeida et al. evaluated the susceptibility of hospital pathogens, including *Staphylococcus aureus* ATCC 25923, methicillin-resistant *Staphylococcus aureus* (MRSA) ATCC 33591, and *Candida albicans* MYA 2876, to nitrochalcone (**9**) (Figure 8). The compound demonstrated activity at MIC of 0.05 mM (15.62 µg/mL) against both bacteria and fungi, with MBC/MIC and MFC/MIC ratios ≤ 4, indicating a significant bactericidal/fungicidal effect. The chemical modification of the chalcone with a nitro group on ring A and a methyl group on ring B resulted in a 16-fold increase in potency compared to the parent chalcone [31].

**Figure 9:**
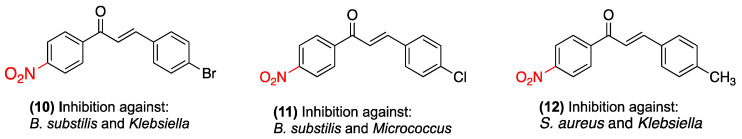
Nitrochalcones **10**–**12** were evaluated in *E. coli*, *S. aureus*, *B. subtilis*, *Klebsiella*, and *Micrococcus* using the disk diffusion method (Bauer-Kirby).

In 2016, Arulkumaran et al. evaluated the antimicrobial activity of a series of 12 nitrochalcones against *Escherichia coli*, *Staphylococcus aureus*, *Bacillus subtilis*, *Klebsiella* spp., and *Micrococcus* spp. using the disk diffusion method (Bauer–Kirby). The results demonstrated that all the evaluated compounds exhibited antimicrobial activity. However, compounds (**10**–**12**) exhibited comparable antibacterial activity against the five strains examined, with active inhibition (20–24 mm) observed in some strains and moderate inhibition (13–19 mm) in others (Figure 9). Compound (**10**) demonstrated active inhibition against *B. subtilis* and *Klebsiella* and moderate inhibition against *E. coli*, *S. aureus*, and *Micrococcus*. Compound (**11**) demonstrated activity against *B. subtilis* and *Micrococcus* and moderate activity against *E. coli*, *S. aureus*, and *Klebsiella*. Finally, compound (**12**) demonstrated activity against *S. aureus* and *Klebsiella*, with moderate activity against *E. coli*, *B. subtilis*, and *Micrococcus* [32].

To provide a comprehensive overview of the antimicrobial potential of the nitrochalcones discussed, Table 3 summarizes their key pharmacological parameters. This summary describes the substitution patterns of each compound, the bacterial and fungal strains evaluated, the observed potency (e.g., minimum inhibitory concentration or inhibition halos), and their main limitations, offering a clear perspective on their viability as antimicrobial agents.

### 3.3. Anti-Inflammatory Activity

**Figure 10:**
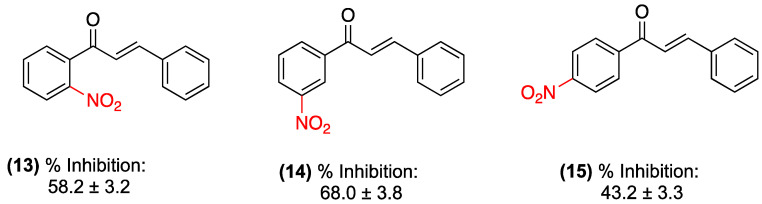
Nitrochalcones **13**–**15** substituted in ring A were evaluated in a carrageenan-induced plantar edema model in rats.

One pharmacological activity of chalcones that has been the focus of research since their discovery is their anti-inflammatory activity. In 2013, our research group, Gómez et al., initiated a study of chalcones bearing nitro substituents. Initially, an anti-inflammatory evaluation of three nitrochalcone isomers (**13**–**15**) containing the nitro group in ring A was conducted. The evaluation employed a carrageenan-induced plantar edema model in rats. The administration was delivered via both oral and intraperitoneal routes. However, the intraperitoneal route yielded superior outcomes. Nitrochalcone (**14**) demonstrated notable performance with a maximum anti-inflammatory protective effect (MAPE) of 68.0% (at 1 h), while (**13**) exhibited the highest area under the curve (AUC) (378.08). Concurrently, chalcones bearing a nitro group at positions 3 and 4 (15) exhibited reduced efficacy compared to meloxicam, the reference pharmaceutical agent (MAPE: approximately 60% in both administration routes). However, nitrochalcone (**13**) showed no statistically significant difference when compared with the reference drug. Therefore, this compound may hold promise, particularly when administered intraperitoneally, exhibiting efficacy comparable to that of meloxicam [33] (Figure 10). The least efficacy, as indicated by its AUC value of 246.78. Nitrated chalcones were less effective than meloxicam (reference drug).

**Figure 11:**
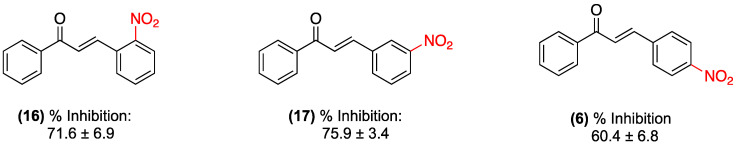
Nitrochalcones substituted in ring B **6**, **16**, and **17** evaluated in a model of carrageenan-induced plantar edema in rats.

Along the same lines of research, our group, Alarcón et al., conducted a study of three additional nitrochalcone isomers (**16**, **17**, and **6**) in ring B. The objective of this study was to analyze the effect of the nitro group in that ring and to compare it with the effects previously studied in ring A. The same carrageenan-induced plantar edema model in rats was utilized for this study. The three nitrochalcones administered intraperitoneally exhibited a significant, dose-dependent protective anti-inflammatory effect (25, 50, 100, and 200 mg/kg), with maximal edema inhibition of approximately 70% at the highest dose (200 mg/kg). The observed effect was of a comparable magnitude to that of the reference drug, meloxicam (10 mg/kg, orally). The study demonstrated that compound (**17**) exhibited a more pronounced anti-inflammatory effect than the other nitrochalcones [34] (Figure 11).

The results of these studies were found to be of interest when compared with prior data from the research group on chalcones with nitro in ring A. In this regard, it is noteworthy that the anti-inflammatory protective effect remained unaltered by the presence of the nitro group. No discrepancy was detected in the course of executing the contrast and Tukey’s test regarding chalcones bearing the nitro group in ring A at a dosage of 200 mg/kg, compounds (**13**–**15**). The Tukey test demonstrated that the position of the nitro group in ring B exhibited comparable values to those of chalcones with the nitro group in ring A. Conversely, these findings suggest that nitrochalcones with the nitro group positioned in ring B exhibit faster absorption rates (1–2 h) compared to chalcones with the nitro group in ring A (3–5 h). Consequently, the latter elicits an anti-inflammatory response more quickly. This finding suggests that the rate of absorption of nitrochalcones administered intraperitoneally is contingent upon the substitution of nitro within the ring structure.

In addition, Hidalgo et al. evaluated the molecules in a supplementary anti-inflammatory model that included dinitrochalcones, i.e., chalcones bearing a nitro group in both ring A and ring B. The base (unsubstituted) chalcone was likewise assessed and utilized as a reference compound. The present study analyzed the effect of the nitro group’s position using in vivo assays and molecular docking.

**Figure 12:**
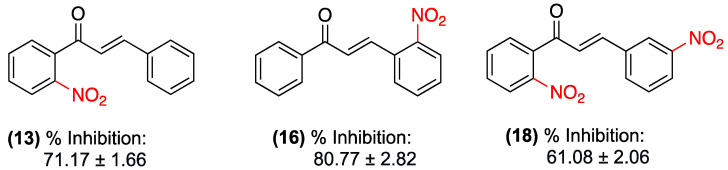
Nitrochalcones **13**, **16**, and **18** were evaluated in the TPA-induced mouse ear edema model.

For the in vivo experiments, the TPA-induced mouse ear edema model was used. The results demonstrated that nitrochalcones with the nitro group in the *ortho* position exhibited the most significant inhibition of the inflammatory process, with the nitro group in the *ortho* position of ring A (**13**) exhibiting an inhibition of 71.17 ± 1.66%; the nitro group in the *ortho* position of ring B (**16**) exhibiting 80.77 ± 2.82%; and the nitro group in the *ortho* position of ring A and in the meta position of ring B (**18**) exhibiting 61.08 ± 2.06%. The activity of these compounds was comparable to, or exceeded, that of the reference drug, indomethacin (71.48 ± 1.62%), and exhibited a statistically significant dose–response relationship (Figure 12).

Molecular docking studies with COX-1 and COX-2 enzymes revealed that compounds (**13**) and (**16**) establish key interactions, primarily hydrogen bonds with residues Arg106 and Arg120. The collective findings indicate that the *ortho* position of the nitro group amplifies the anti-inflammatory activity, a phenomenon that may be attributable to electronic effects that augment the reactivity of the enone system and facilitate interaction with the active sites of COX-1 and COX-2 [4].

**Figure 13:**
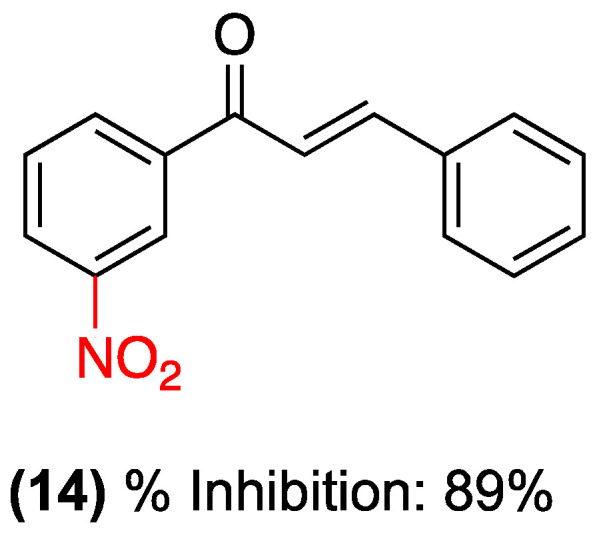
Nitrochalcone **14** is evaluated as a model of albumin denaturation inhibition.

In addition to studies conducted in our laboratory, other experimental models have been used to evaluate the anti-inflammatory effects of chalcones. In 2013, Balasubramanian et al. [35] conducted a preliminary study to determine the anti-inflammatory activity of chalcones substituted in ring A, including nitrochalcone (**14**) (Figure 13). The findings indicated that this compound exhibited an effect analogous to that of the reference drug, with approximately 89% inhibition at 30 μg/mL in a model of albumin denaturation inhibition, originally proposed by Mizushima [36] and subsequently modified. The preceding evidence substantiates the notion that nitrochalcones warrant further investigation into their anti-inflammatory properties, thereby facilitating a deeper understanding of their molecular mechanisms.

To provide a comprehensive overview of the anti-inflammatory potential of the nitrochalcones discussed, Table 4 summarizes their key pharmacological parameters. This summary describes specific substitution patterns, biological models evaluated, observed potency or efficacy, routes of administration, and their performance compared to standard reference drugs, offering a clear comparative perspective on their therapeutic viability.

### 3.4. Analgesic Activity

**Figure 14:**
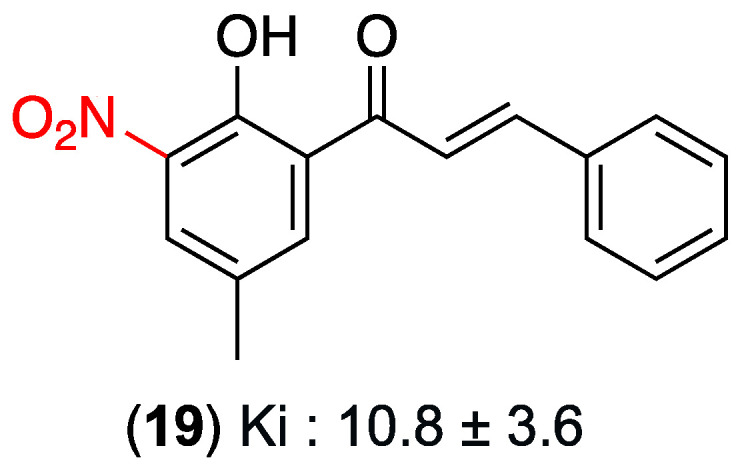
Nitrochalcone **19** with antinociceptive effect.

In addition to their anti-inflammatory activity, nitrochalcones have been assessed for their analgesic potential. In 2019, Higgs’ research group evaluated a series of synthetic chalcone derivatives for their analgesic potential. The focus of this evaluation was on the interaction of the derivatives with the μ-opioid receptor and their effects in acute pain models in mice. Among the synthesized compounds, 5′-methyl-2′-hydroxy-3′-nitrochalcone (**19**) was particularly noteworthy for its notable antinociceptive activity, demonstrating a high degree of affinity for the μ-opioid receptor: Ki = 10.8 ± 3.6 μM (moderate affinity in the micromolar range) and peripheral antinociceptive activity (contortion test) at 10 mg/kg significantly inhibited contortions (Figure 14). The observed effect was found to be dose-dependent, ranging from 0.3 to 30 mg/kg, with the highest administered dose resulting in 96.1% inhibition. In the context of central antinociceptive activity, as measured by the hot plate test, it was reported that administration of 30 mg/kg resulted in a significant increase in response latency to the thermal stimulus. This finding suggests the presence of a central mechanism underlying the observed effects. Locomotor activity and rotarod tests confirmed that the observed analgesic effect was not attributable to sedation or motor toxicity. The study demonstrated no substantial cytotoxicity in the SH-SY5Y neuroblastoma cell line at pertinent concentrations (74.8% viability at 20 μM) [37].

**Figure 15:**
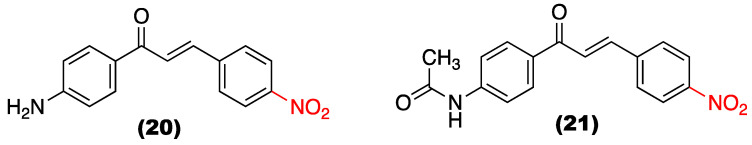
Nitrochalcones **20** and **21** were evaluated in acute pain mouse models.

Concomitantly, in 2018, Rocha et al. appraised the analgesic capacity of two nitrochalcones (**20**) and (**21**) in murine models of acute and persistent pain. The investigation revealed that both nitrochalcones significantly inhibited carrageenan-induced mechanical hypersensitivity, both when administered intraperitoneally and when administered orally. The efficacy profile of the nitrochalcones was comparable to that of indomethacin. The efficacy of the compounds in question was also demonstrated in various models of persistent pain, including the inflammatory (CFA), neuropathic (partial sciatic nerve ligation—PSNL), and cancer (B16F10 melanoma) pain models. Among these models, compound (**20**) exhibited the greatest efficacy in the cancer model. A reduction in leukocyte migration and myeloperoxidase (MPO) activity was reported, as well as an inhibition of proinflammatory cytokine levels (IL-1β and TNF) in inflamed tissue and in LPS-stimulated macrophages. (**20**) exhibited the capacity to curtail the hypersensitivity induced by IL-1β, TNF, epinephrine, and PGE_2_, while (**21**) demonstrated an absence of efficacy against PGE_2_. The observed effects did not extend to IL-10 or KC/CXCL1 levels, suggesting a selective mechanism involving specific proinflammatory pathways (Figure 15). The substances in question were found to comply with Lipinski and Veber’s rules, indicating good oral bioavailability and permeability [38]. Table 5 summarizes the pharmacological studies conducted for compounds **19** to **21**.

### 3.5. Antiparasitic Activity

**Figure 16:**
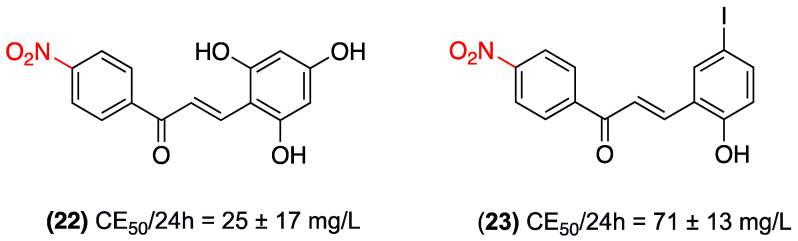
Nitrochalcones **22** and **23** were evaluated against the nematode *Meloidogyne incognita*.

In the context of antiparasitic potential, in 2015, Caboni et al. investigated the nematicide efficacy of nitrochalcones (**22**) and (**23**) against the root knot nematode, *Meloidogyne incognita*. The study revealed that both nitrochalcones demonstrated significant paralyzing activity on second-stage juveniles, with an EC_50_/24 h = 25 ± 17 mg/L for (**22**) and 71 ± 13 mg/L for (**23**) (EC_50_/24 h). These findings substantiate the nitro group as a pivotal component of the pharmacophore (Figure 16). The presence of a nitro group at position 4 of the chalcone is associated with greater nematicide activity, and its structural modification in chalcones maintains or modulates this activity, depending on the substituents of ring B [39].

**Figure 17:**
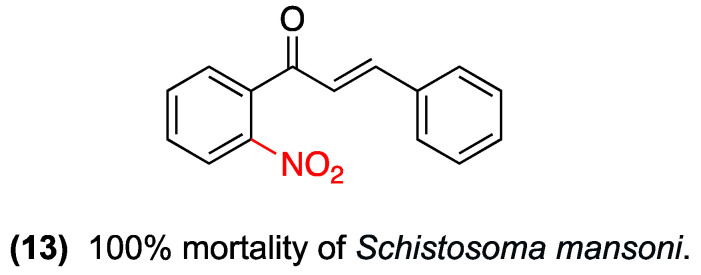
Nitrochalcone **13** was evaluated against *Schistosoma mansoni*.

Similarly, Marcovicz et al. evaluated nitrochalcone (**13**), which showed potent antischistosomal activity in vitro against adult *Schistosoma mansoni*. The study demonstrated that the survival assay resulted in 100% mortality of the worms at all concentrations evaluated (25, 50, 100, and 200 μg/mL) after only 2 h of incubation. With regard to its sublethal effects, at a concentration of 25 μg/mL, (**13**) induced the separation of 60% of worm pairs at 6 h, an effect sustained up to 72 h. Furthermore, the study found that (**13**) completely suppressed oviposition (0%) at all concentrations and times observed (up to 72 h), compared to the control group, which showed 30% oviposition at 6 h and 60% at 24 h. Therefore, the study confirms that (**13**) exhibits marked antiparasitic activity, characterized by rapid lethality and significant inhibitory effects on the parasite’s motility, mating, and reproduction (Figure 17) [40]. Table 6 summarizes the two studies on the analgesic evaluation of nitrochalcones.

### 3.6. Antifungal Activity

**Figure 18:**
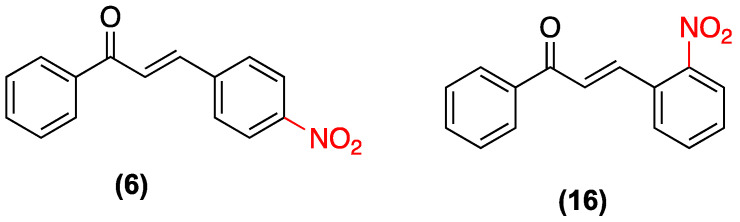
Nitrochalcones **6** and **16** evaluated in dermatophytes.

In the search for new antifungal agents, chalcones have emerged as promising structures due to their structural simplicity and synthetic versatility. In this regard, the study by López et al. in 2001 systematically evaluated 41 chalcone derivatives, among which nitrochalcones exhibited an interesting pharmacological profile. The group revealed that substitution with nitro groups in the B ring of the chalcone significantly influences antifungal activity (Figure 18). Specifically, (**6**) demonstrated significant antifungal activity against dermatophytes, exhibiting higher potency against the fungus *Epidermophyton floccosum*, with MIC values as low as 0.25 µg/mL, surpassing those of the unsubstituted chalcone. In contrast, (**16**) exhibited a significant decrease in activity; however, its effect was superior to that of most compounds that do not possess the nitro group (Table 7) [41].

**Figure 19:**
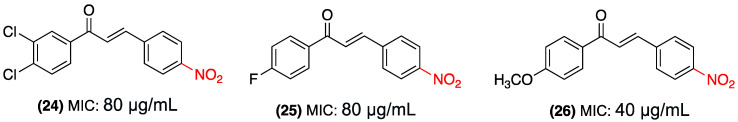
Nitrochalcones **24**–**26** evaluated for their antifungal activity.

In a 2012 study by Tristao et al., the antifungal potential of a series of 23 synthetic chalcones, including five nitrochalcones, was systematically evaluated. The results of this study underscore the significance of this subgroup, as it demonstrates moderate antifungal activity and a favorable toxicity profile in cell lines. Among the five nitrochalcones evaluated, three exhibited significant antifungal activity: compound (**24**) demonstrated activity against *Aspergillus niger* (MIC = 80 µg/mL) and *Trichophyton rubrum* (MIC = 100 µg/mL). It was demonstrated that compound (**25**) was active against *A. niger*, with an MIC of 80 µg/mL. Finally, nitrochalcone (**26**) is active against *Trichophyton mentagrophytes* with an MIC of 40 µg/mL (Figure 19) [42].

**Figure 20:**
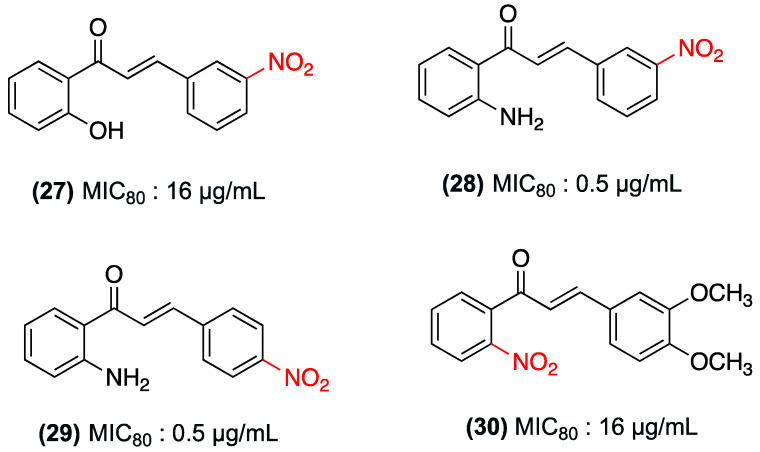
Nitrochalcones **27**–**30** with antifungal effect evaluated against *T. rubrum*.

In a study by Zhang et al. in 2018, 25 substituted chalcones, including nitrochalcones, were designed and synthesized to evaluate their antifungal activity against the dermatophyte *Trichophyton rubrum*, the main etiological agent of dermatophytosis. The study’s findings demonstrated that nitrochalcones (**27**–**30**) exhibited noteworthy antifungal properties. Compounds (**28**) and (**29**) exhibited potent activity, with an MIC_80_ of 0.5 µg/mL, comparable to that of the positive control fluconazole (MIC_80_ = 0.25 µg/mL). Furthermore, compounds (**27**) and (**30**) demonstrated moderate activity (MIC_80_ = 16 µg/mL) (Figure 20). The authors observed that the combination of a 2′-amino group on ring A with a nitro substituent on ring B increases antifungal potency. This indicates that substituting the 2′ position of ring A with amino groups yields superior results compared to substituting with hydroxyl groups [43]. Table 8 summarizes studies on nitrochalcones evaluated for antifungal activity.

### 3.7. Antileishmanial Activity

**Figure 21:**
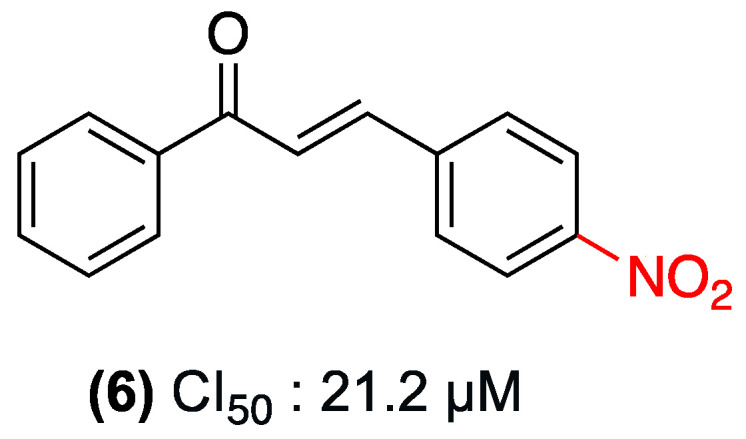
Nitrochalcone **6** with activity against *Leishmania amazonensis*.

A 2020 study by Assolini et al. examined the antiparasitic potential of various chalcone derivatives. Among these derivatives, nitrochalcone (**6**), distinguished by the presence of a nitro substituent in the *para* position of ring B, demonstrated notable significant activity against *Leishmania amazonensis*, the causative agent of American cutaneous leishmaniasis. It has been demonstrated that nitrochalcone (**6**) exerts a direct effect on *promastigote* forms, with a mean inhibitory concentration (IC_50_) of 21.2 µM (Figure 21). Mechanistic studies have revealed that this compound induces a late apoptotic death process, accompanied by increased production of reactive oxygen species (ROS), mitochondrial membrane depolarization, phosphatidylserine exposure, and significant morphological alterations, including a reduction in the parasite’s cell volume [44].

**Figure 22:**
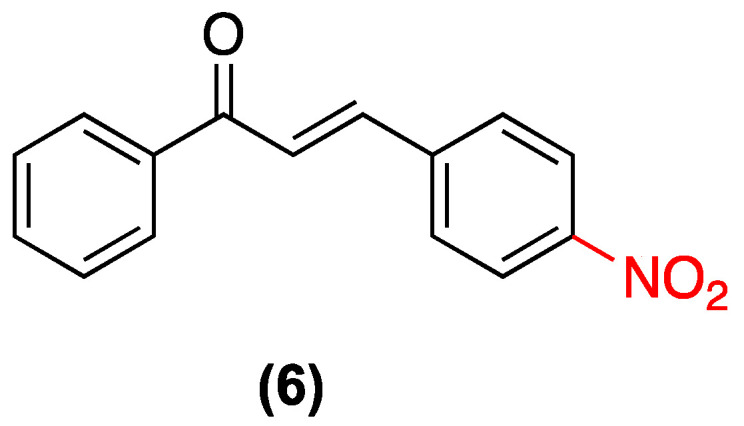
Nitrochalcone **6** with an effect against the *promastigote* of *L. infantum* and *L. amazonensis*.

In 2025, Barreiros and colleagues also conducted studies on the antileishmanial activity of nitrochalcone (**6**). In this study, beeswax-based solid lipid nanoparticles were developed as a delivery system for the compound (**6**), which exhibits leishmanicidal activity. The mechanism of action of this compound involves disruption of mitochondrial function, leading to inhibition of macromolecular biosynthesis and an increase in reactive oxygen species (ROS) within the *Leishmania* parasite (Figure 22) [45].

In vitro assays revealed that free nitrochalcone (**6**) caused a significant reduction in the viability of the promastigote forms of L. infantum and L. amazonensis, whereas the lipid nanoparticles containing compound (**6**) did not exhibit a direct toxic effect on these parasitic stages. The lipid nanoparticle formulations with (**6**) exhibited no evidence of cellular toxicity against J774.1 macrophages at concentrations below 10 µM, contrasting with the observations reported in previous studies with free 4-nitrochalcone. This finding suggests a reduction in cellular toxicity with the lipid nanoparticle formulations. The authors hypothesize that lipid nanoparticles function as controlled release systems, engineered to be internalized by macrophages (the host cells of the parasite in their amastigote form). The sustained release of the compound within the infected cell would result in an enhancement of the local concentration of (**6**) in the parasitic niche, thereby potentially enhancing therapeutic efficacy in a targeted manner [45].

In 2018, Sousa-Batista et al. evaluated an innovative single-dose strategy for localized cutaneous leishmaniasis. The strategy utilized biodegradable microspheres made of polylactic-co-glycolic acid copolymer as a sustained-release system for synthetic nitrochalcone (**31**) [46].

**Figure 23:**
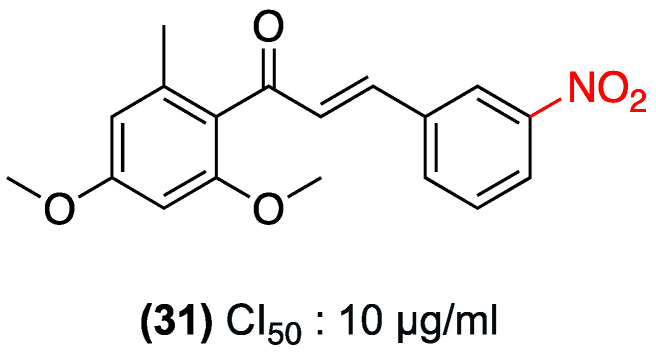
Nitrochalcone **31** with activity against cutaneous leishmaniasis.

In vitro studies demonstrated that murine macrophages efficiently internalized microspheres of (**31**) (particles < 6 µm) within 3 h. Encapsulation was found to be an effective strategy for maintaining the high leishmanicidal activity of compound (**31**) against intracellular amastigotes of *Leishmania amazonensis* (IC_50_ of approximately 10 µg/mL). This approach led to a significant reduction in the compound’s toxicity toward macrophages, thereby improving its safety profile (Figure 23). Moreover, neither the free (**31**) nor the microspheres induced oxidative mechanisms (nitric oxide and reactive oxygen species) in macrophages, thereby confirming that their antiparasitic action is direct and not mediated by host immune system stimulation [46]. Table 9 summarizes studies on nitrochalcones evaluated for antileishmanial activity.

### 3.8. Cytotoxic Activity

**Figure 24:**
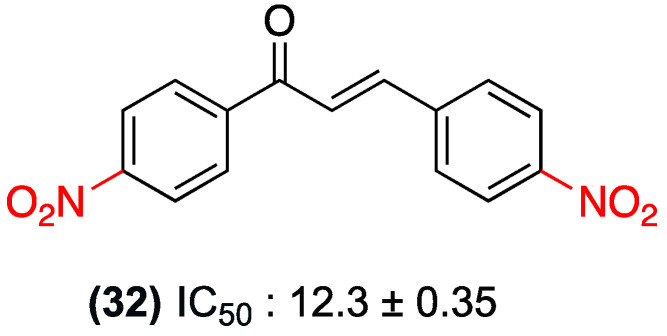
Dinitrochalcone **32** evaluated against the human nasopharyngeal carcinoma cell line KB.

A number of studies have been conducted on nitrochalcones, with a particular emphasis on their cytotoxic properties. The study by Qiu et al. in 2012 is a systematic contribution to this field, synthesizing and evaluating 10 derivatives of 4-nitrochalcone against the human nasopharyngeal carcinoma cell line KB. This study allowed clear structure-activity relationships to be elucidated and the potency of these compounds to be ranked. Compound (**32**) was identified as the most potent, with an IC_50_ of 12.3 µM, surpassing the positive control 5-fluorouracil (IC_50_ = 16.9 µM) (Figure 24) [47].

**Figure 25:**
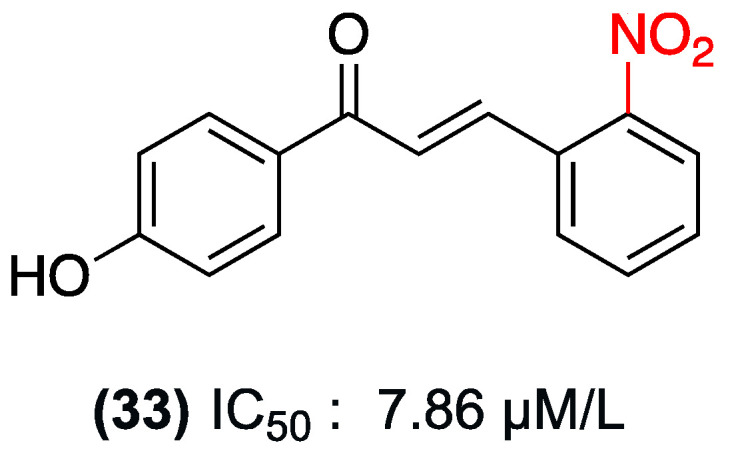
Nitrochalcone **33** is evaluated in a rhabdomyosarcoma cell line.

Continuing with the study of the cytotoxic activity of nitrochalcones, Abbas Al-Kelaby and colleagues conducted experiments in 2016 on a rhabdomyosarcoma cell line. Rhabdomyosarcoma is a soft tissue sarcoma that is commonly diagnosed in children. In these experiments, the effect of compound (**33**) was compared with standard chemotherapeutic drugs such as doxorubicin and methotrexate. The modulation of compound (**33**) in combination with a monoterpene was also investigated (Figure 25). The nitrochalcone derivative (**33**) demonstrated substantial inhibition of cell proliferation in rhabdomyosarcoma, with a CI_50_ of 7.86 µM [48].

In comparison, doxorubicin and methotrexate exhibited lower IC_50_ values (0.553 and 0.8708 µg/mL, respectively). However, statistically significant differences were not observed between compound (**33**) and these drugs (*p* > 0.05), suggesting that (**33**) possesses comparable anticancer potency.

**Figure 26:**
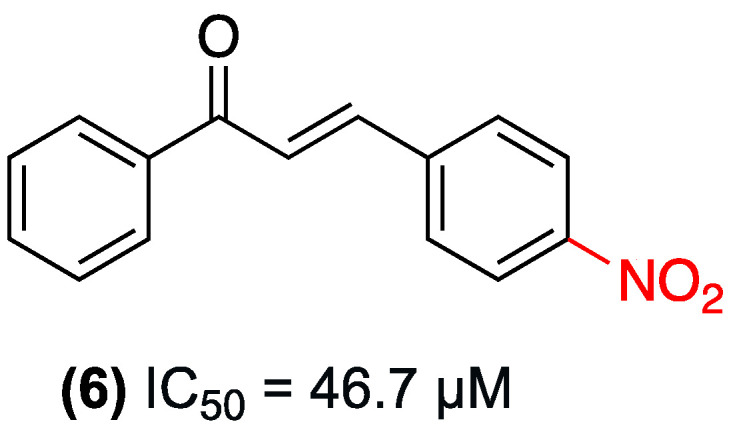
Nitrochalcone **6** evaluated in HeLa cells.

In a related study, Carpio et al. also evaluated the cytotoxic activity of 4-nitrochalcone (**6**) in 2019, both in its free form and encapsulated in folic acid-functionalized poly(methyl methacrylate) nanocapsules. The free compound (**6**) exhibited moderate, dose-dependent toxicity in HeLa cells, with an IC_50_ value of 46.7 µM. However, its encapsulation in the functionalized nanostructured system significantly enhanced its effect, reducing cell viability by approximately 30% and 63% at concentrations of 15 and 30 µM, respectively. These values are significantly higher than those observed with free (**6**) at the same concentrations (Figure 26). This increase in potency was attributed to greater internalization mediated by folate receptors overexpressed in tumor cells. Furthermore, both the free (**6**) and the nanocapsulated formulation exhibited a selective profile, demonstrating no significant toxicity in non-tumor NIH3T3 fibroblasts or human erythrocytes up to a concentration of 50 µM [49].

**Figure 27:**
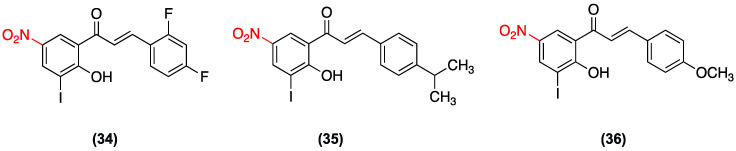
Nitrochalcones **34**–**36** were evaluated for cytotoxicity in A549 and MCF-7 cell lines.

In 2024, Mphahlele et al. evaluated a series of fourteen 2′-hydroxy-3′-iodo-5′-nitrochalcone derivatives for their cytotoxic activity in lung (A549) and breast (MCF-7) cancer cell lines, as well as their potential to inhibit VEGFR-2 tyrosine kinase. The compounds exhibited moderate toxicity compared with the reference drugs (Figure 27). In A549 cells, the IC_50_ values ranged from 13.36 ± 0.29 µM to 52.16 ± 2.40 µM. Compound (**34**)**,** which was substituted with 2,4-difluorophenyl, exhibited the highest activity, with an IC_50_ value of 13.36 ± 0.29 µM. In MCF-7 cells, the IC_50_ values ranged from 11.98 ± 0.09 µM to 30.66 ± 1.15 µM. Compounds (**35**) with an IC_50_ of 11.98 ± 0.09 µM and (**36**) with an IC_50_ of 12.08 ± 0.54 µM were identified as the most potent. It is noteworthy that all compounds exhibited significantly reduced toxicity in non-cancerous HEK293-T cells (see Table 10) [50].

To provide a comprehensive overview of the cytotoxic potential of the discussed nitrochalcones, Table 11 summarizes their key pharmacological parameters. This summary outlines the evaluated cancer cell lines, the observed half-maximal inhibitory concentrations (IC_50_), and specific mechanistic strategies, including potential VEGFR-2 inhibition and the use of folate-functionalized nanocapsules for targeted delivery. Furthermore, it highlights the selectivity of these compounds towards malignant cells compared to non-tumorigenic cell lines.

### 3.9. Miscellaneous

This section presents studies on other pharmacological activities of nitrochalcones that have been less studied or less reported in the literature. For instance, the antiacetylcholinesterase activity is relevant to the search for palliative agents for Alzheimer’s disease, where inhibition of this enzyme is an established therapeutic strategy to increase acetylcholine levels in the brain.

In 2014, Ibrahim et al. evaluated the inhibitory activity of acetylcholinesterase using two assays: thin-layer chromatography (TLC) bioautography and a quantitative microplate assay (Ellman method) [51].

**Figure 28:**
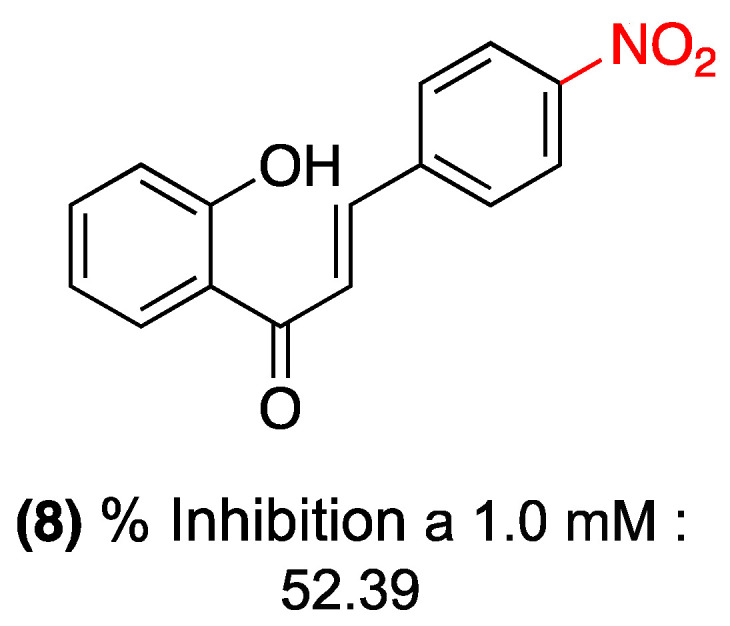
Nitrochalcone **8** with acetylcholinesterase inhibitory activity.

In the microplate assay, nitrochalcone (**8**) demonstrated moderate inhibition of acetylcholinesterase. The study reported 52.39% inhibition at a concentration of 1.0 mM. However, at a concentration of 0.1 mM, no detectable activity was observed, suggesting that the compound’s potency is moderate to low compared to the positive control (Figure 28).

In the TLC assay, no detectable activity was observed for this compound, suggesting that the microplate assay may be more sensitive for quantifying its effect. The authors posit that the moderate activity of compound (**8**) could be attributed to the presence of the nitro group (–NO_2_) in the *para* position of the chalcone B ring [51].

**Figure 29:**
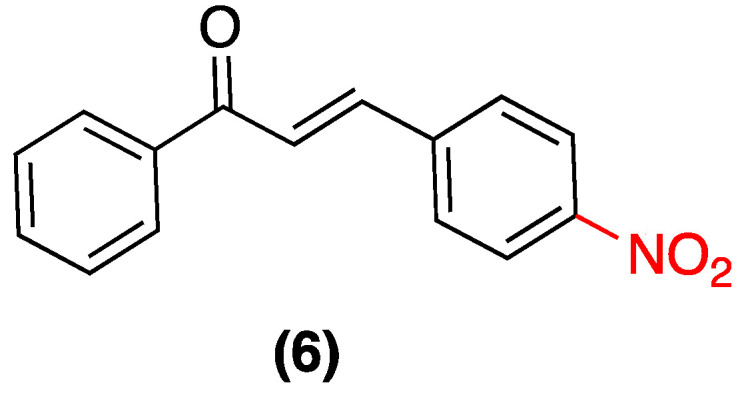
Nitrochalcone **6** with inhibitory effect on human monoamine oxidase B.

Oh et al. (2019) studied 4-nitrochalcone (**6**), which demonstrated a remarkable dual-targeting pharmacological profile (Figure 29). The study revealed that the compound exhibited potent and selective inhibition of human monoamine oxidase (hMAO) B with an IC_50_ of 0.066 µM and a high selectivity index against hMAO-A (SI = 137.9). Additionally, it demonstrated significant inhibition of acetylcholinesterase (AChE) with an IC_50_ of 1.25 µM, the lowest among the compounds evaluated in the study. This is interesting because nitrochalcone (**6**) may serve as a fundamental structural element in the development of a potential dual-target neuroactive agent for the treatment of neurodegenerative diseases such as Alzheimer’s [52].

**Figure 30:**
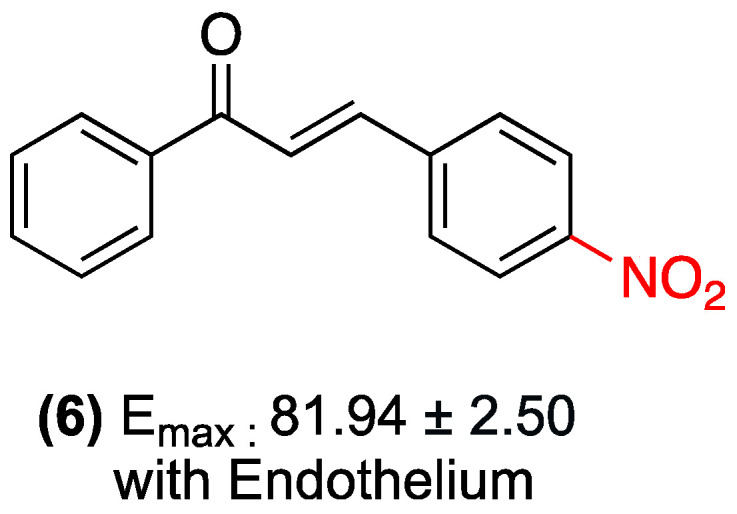
Nitrochalcone **6** with vasorelaxant effect.

In a study conducted by Hidalgo et al., the vasorelaxant activity of a series of nitrochalcones was evaluated in an isolated organ model (aortic rings). The study found that compound (**6**) (nitro group in the para position of ring B), with a maximum vasorelaxant effect of 81.94%, was the compound with the greatest vasorelaxant effect. The authors propose that the mechanism of action mainly operates via the nitric oxide pathway, as confirmed by its interaction with eNOS and inhibition with L-NAME (Figure 30) [4].

## 4. Discussion

### Pharmacological Relevance of Nitrochalcones as Multitarget Scaffolds

A comprehensive analysis of the extant literature indicates that nitrochalcones constitute a particularly versatile subclass of chalcone derivatives. The incorporation of the nitro group (–NO_2_) exerts a pronounced effect on the physicochemical properties and biological activity of these compounds. The combination of the α,β-unsaturated pharmacophore with a strongly electroattractive substituent generates a molecular framework capable of interacting with multiple biological targets. This molecular framework may explain the broad spectrum of pharmacological activities of these compounds.

The biological effects of nitrochalcones include cytotoxicity, anti-inflammatory, antiparasitic, and antimicrobial activities. From a mechanistic perspective, several of the biological effects of nitrochalcones appear to converge on common molecular pathways. These pathways include modulation of oxidative stress, mitochondrial dysfunction, induction of apoptosis, and inhibition of enzymes. The nitro group has been shown to play a dual role in these processes, acting as an electron modulator that increases the enone system’s electrophilic character. This, in turn, facilitates Michael-type interactions with nucleophilic residues of proteins. Additionally, nitrochalcones have been observed to function as redox-active units capable of participating in intracellular bioreduction processes. These processes have the potential to generate reactive oxygen species (ROS). The observations are consistent with the multitarget behavior previously reported for nitrochalcones, thereby underscoring their potential as privileged scaffolds in medicinal chemistry.

In the field of cancer research, nitrochalcones have exhibited selective toxicity against a diverse array of cancer cell lines, encompassing colon, breast, esophageal, and lung cancer. It has been demonstrated that several derivatives can induce apoptosis via ROS-mediated pathways, alter cell cycle progression, and modulate critical signaling cascades, including mTOR and GSK3β, as well as anti-apoptotic proteins such as MCL-1. Notably, several studies have documented significant tumor growth inhibition in vivo without apparent systemic toxicity, suggesting that nitrochalcones may offer a favorable balance between efficacy and safety when compared to conventional chemotherapeutic agents [26,27,28,29].

In addition, the anti-inflammatory activity of nitrochalcones has been demonstrated in a variety of experimental models. Data from carrageenan-induced edema and TPA-induced ear edema models, supplemented by molecular docking studies, suggest that the position of the nitro group influences not only the magnitude of the biological response but also pharmacokinetic parameters, such as absorption and onset of action. In this context, the nitro group appears to act as both a pharmacodynamic and pharmacokinetic modulator, enhancing interactions with key inflammatory enzymes such as COX-1 and COX-2 and influencing the temporal profile of the anti-inflammatory effect.

**Figure 31:**
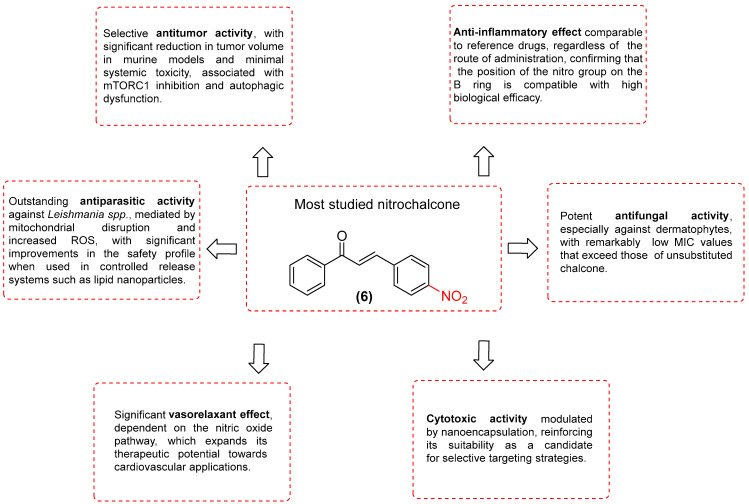
Most studied nitrochalcone position (**6**).

Within this broad pharmacological landscape, 4-nitrochalcone (**6**) emerges as a particularly relevant molecule due to its proven biological activity, demonstrated across a variety of experimental models. The extant research, both in vitro and in vivo studies, indicates that the compound exhibits anticancer, anti-inflammatory, antiparasitic, antifungal, neuroactive, and vasorelaxant properties. This evidence suggests that the compound in question possesses a multifunctional pharmacological profile (Figure 31). However, we recognize that many studies remain to be conducted before we can affirm that 4-nitrochalcone is a privileged scaffold. Its capacity to interact with a variety of molecular targets, in conjunction with its modifiable physicochemical properties and compatibility with advanced drug delivery systems, underscores its potential as a lead structure for future drug discovery strategies.

The analysis of the structure–activity relationship presented in this study indicates that the biological activity of nitrochalcones is governed by a complex interaction of electronic, steric, and conformational factors, in addition to the presence of the nitro group. The strong electron-withdrawing nature of the nitro substituent significantly alters the electronic distribution of the conjugated system, increasing the electrophilicity of the α,β-unsaturated enone and, therefore, its propensity to participate in Michael-type interactions with biological nucleophiles, such as cysteine residues in regulatory proteins [53].

Furthermore, the nitro group has been observed to participate in intracellular redox processes, thereby generating ROS or serving as an electron acceptor within enzymatic systems. This redox behavior provides a plausible mechanistic explanation for the multitarget pharmacological effects observed in nitrochalcones, particularly in anticancer and antiparasitic contexts, where oxidative stress plays a central role.

A critical determinant of nitrochalcone activity is the position of the nitro group within the aromatic rings. It has been established that the *para* pattern is associated with increased biological activity. The observed effect can be attributed to the optimal conjugation with the α,β-unsaturated system. This conjugation amplifies the electron-withdrawing influence of the nitro group and stabilizes the conjugated π system. Conversely, nitro substitution in ring A tends to exert a more indirect modulatory effect, influencing selectivity, physicochemical properties, and interactions with biological targets, rather than intrinsic potency.

Substitutions in *ortho* and *meta* positions introduce additional steric and electronic effects that have the capacity to enhance or diminish biological activity depending on the specific molecular context. *Ortho* substitution, for instance, has been shown to induce conformational distortions that can affect conjugation and target binding affinity. *Meta* substitution, on the other hand, has been observed to generally result in weaker electronic communication with the enone system. These observations suggest that the biological behavior of nitrochalcones results from a delicate balance between conjugation, steric hindrance, and electronic polarization within the molecular framework (Table 12).

Taken together, these SAR findings reinforce the notion that nitrochalcones can be regarded as a tunable structural platform for drug design. By strategically modulating the substitution pattern and nature of substituents on the aromatic rings, it is possible to optimize both potency and selectivity while minimizing the potential toxicity associated with nitroaromatic compounds.

## 5. Limitations and Prospects

Notwithstanding the encouraging pharmacological profile of nitrochalcones, their development into clinically viable drug candidates is impeded by several limitations. The majority of studies documented in the extant literature are based on in vitro models, which, while providing valuable mechanistic information, do not fully reflect the complexity of in vivo biological systems. Moreover, the paucity of exhaustive pharmacokinetic, toxicological, and metabolic investigations impedes the assessment of nitrochalcones’ safety profile and drug-like properties.

A further significant constraint pertains to the heterogeneity of experimental designs, encompassing variations in biological models, test conditions, and evaluation criteria. This variability complicates the establishment of direct comparisons between studies and undermines the robustness of SAR conclusions. Furthermore, publication bias may overestimate the pharmacological potential of nitrochalcones, as negative or inconclusive results are less frequently reported.

From a medicinal chemistry perspective, future research should prioritize systematic SAR studies based on libraries of structurally coherent compounds, coupled with integrated ADME/Tox assessments and mechanistic validation using orthogonal experimental approaches. Likewise, the dual nature of the nitro group—as both a pharmacophoric and potentially toxic unit—must be addressed with caution through rational design strategies that maximize therapeutic efficacy and minimize adverse effects.

In addition to the challenges mentioned above, the physicochemical properties of nitro-substituted chalcones must be carefully considered during their preclinical evaluation. Nitrochalcones often exhibit limited aqueous solubility, which not only hinders their bioavailability in vivo but can also lead to aggregation or precipitation of the compound in vitro assay media, potentially resulting in erratic or false-negative data. Furthermore, the extended conjugated system of these molecules, coupled with the nitro group, often confers intense intrinsic coloration (typically yellow to orange) and, in some cases, fluorescent properties. These optical characteristics can significantly interfere with standard spectroscopy- or fluorescence-based biological readings, such as the widely used [3-(4,5-dimethylthiazol-2-yl)-2,5-diphenyltetrazolium bromide] (MTT) or resazurin reduction assays [54]. This interference can generate artifacts in the assay—such as the internal filter effect or background signal absorption/emission—leading to overestimation or underestimation of the compound’s true pharmacological efficacy [55].

To mitigate these limitations and distinguish true biological activity from assay interference, it is crucial to implement orthogonal validation strategies [56]. Researchers should strongly consider replacing colorimetric assays with label-free technologies, luminescent assays (e.g., CellTiter-Glo), or morphological assessments. Additionally, the systematic use of appropriate blank controls (a compound in a medium without cells/enzymes) is mandatory to subtract background noise. Likewise, as highlighted in several studies within this review, the use of targeted delivery systems—such as encapsulation in solid lipid nanoparticles, microspheres, or functionalized nanocapsules—represents a highly effective approach to simultaneously address the poor aqueous solubility of nitrochalcones and mask their optical interference, thereby maximizing their therapeutic potential.

## 6. Conclusions

Nitrochalcones are distinguished by their remarkable pharmacological versatility and multi-target behavior. The presence of the nitro group has been identified as a critical structural element, as it modulates the reactivity of the α,β-unsaturated system and promotes molecular interactions associated with redox processes, cell signaling, and enzyme inhibition. This observation suggests a possible explanation for the observed diversity of biological activities. Among the various compounds examined in this study, 4-nitrochalcone (6) has been studied in vitro and in vivo, demonstrating antitumor, anti-inflammatory, antiparasitic, antifungal, and vasorelaxant properties. Therefore, its ability to act on multiple molecular targets is very noteworthy. However, further studies are needed to confirm whether this nitrochalcone could be a privileged molecule. Nevertheless, this review highlights notable limitations, in particular the paucity of pharmacokinetic, toxicological, and translational studies that would facilitate a comprehensive assessment of the safety and clinical viability of nitrochalcones.

## Figures and Tables

**Table 1:** Some reviews of natural and synthetic chalcones.

Chalcone/Source	Biological Activities	Future Developments	Reference
Highly functionalized chalcones/Natural	Multiple in vitro and in vivo pharmacological activities	Comprehensive research investigations, especially in preclinical and clinical studies.	Samota et al., 2024 [15]
	Multiple pharmacological activities, including SAR studies and the mechanism of action		Adhikari et al., 2025 [14]
Highly functionalized chalcones/Synthetic and natural	Cancer	Synthesis and structural optimization, along with desired potency, selectivity, in vivo efficacy, and chalcone complexes, are desirable	Karthikeyan et al., 2015 [17]
	Antiviral		Elkhalifa et al., 2021 [18]
	Pharmacological activities such as antibacterial, antifungal, antitubercular, antioxidant, and antimalarial		Mezgebe et al., 2023 [19]
	Multiple pharmacological activities, including molecular mechanisms and clinical evidence		Salehi et al., 2021 [20]
	Antidiabetic		Kaushal et al., 2021 [21]
Bis-chalcones/Synthetic	Anti-inflammatory	Deep analysis of the structure-activity relationship (SAR)	Pereira et al., 2023 [22]
Flurochalcones/Synthetic	Multiple pharmacological activities (particularly antibacterial, antiviral, and anticancer)	Mechanistic studies, pharmacokinetic profiling, and clinical application	Kubiak et al., 2025 [23]
Nitrochalcones/Synthetic	Multiple pharmacological activities		This Work

**Table 2:** Summary of Anticancer Activity of Nitrochalcones.

Compound	Substitution Pattern	Target/Pathway	Biological Model	Potency	Selectivity	Key Limitations
(**1–3**)	4-nitro (Ring B)	Acts as an electron acceptor, inducing selective apoptosis (instead of necrosis).	Human colon cancer cell lines (HT-29, LS180, LoVo, and LoVo/DX).	IC_50_: 0.96–2.24 μg/mL (for compound 1).	Superior activity against resistant cell lines (LoVo/DX).	The underlying mechanism remains hypothetical; there is a lack of in vivo validation.
(**4**)	4-nitro (Ring B)	Dose-dependent apoptosis (caspase-7, c-PARP, Bax/Bcl-2); suppression of epithelial–mesenchymal transition (EMT); GSK3β inhibition.	Breast cancer stem cells (MCF-7SC).	IC_50_: 1.33 μM.	Strongly reduces the cancer stem cell population (CD44+/CD24−); favorable oral absorption.	Poor aqueous solubility.
(**5**)	4-nitro (Ring B)	Reactive oxygen species (ROS) generation, G2/M cell cycle arrest, and apoptosis.	Esophageal squamous cell carcinoma (ESCC: KYSE-450 and Eca-109); mouse xenograft model.	IC_50_: 4.97 µM (KYSE-450) and 9.43 µM (Eca-109).	Favorable toxicity profile (reduces tumor volume without affecting body weight).	High cytotoxicity toward normal cells and potential off-target effects.
(**6**)	4-nitro (Ring B)	Dysfunctional autophagy (increase in LC3-II); mTORC1 inhibition; alteration of tumor energy metabolism.	In vivo Ehrlich carcinoma model; breast cancer cell lines (MCF-7, MDA-MB-231).	44–58% reduction in tumor volume in vivo.	Preferential toxicity in MCF-7 (Selectivity Index = 3.68) with minimal damage to non-tumoral cells (HB4a).	Scarcity of pharmacokinetic or toxicological studies for clinical use.
(**7**)	2-nitro (Ring B)	Inhibitor of the anti-apoptotic protein MCL-1; plasma membrane rupture and apoptosis.	HCT116 cells (colon cancer).	IC_50_: 15.18 μM; apoptosis at 7.79 μM.	Inhibits tumor resistance by docking into the active site of MCL-1.	Lack of in vivo studies supports this mechanism.

**Table 3:** Summary of Antimicrobial Activity of Nitrochalcones.

Compound	Substitution Pattern	Target/Pathway	Microbial Model	Potency	Selectivity	Key Limitations
(**8**)	4-nitro (Ring B).	Not explicitly reported.	*Enterococcus faecalis*, *Bacillus cereus*, *Klebsiella pneumoniae*, *Pseudomonas aeruginosa*, *Aspergillus fumigatus*, *Candida glabrata*.	MIC: 125 µg/mL (Gram+); 500 µg/mL (Gram−); 250 µg/mL (fungi).	Moderate activity against Gram-positive bacteria.	Low effectiveness relative to standard control.
(**9**)	4-nitro (Ring A)	Not explicitly reported.	Hospital pathogens: *Staphylococcus aureus* ATCC 25923, MRSA ATCC 33591, and *Candida albicans* MYA 2876.	MIC: 0.05 mM (15.62 µg/mL).	Significant bactericidal/fungicidal effect (MBC/MIC and MFC/MIC ratios ≤ 4).	Not explicitly reported.
(**10**–**12**)	4-NO_2_ (Ring A).	Not explicitly reported.	*Escherichia coli*, *Staphylococcus aureus*, *Bacillus subtilis*, *Klebsiella* spp., and *Micrococcus* spp.	Active inhibition (20–24 mm) and moderate inhibition (13–19 mm).	Varying profiles: Compound 10 is active against *B. subtilis* and *Klebsiella*; 11 against *B. subtilis* and *Micrococcus*; 12 against *S. aureus* and *Klebsiella*.	Assayed using the disk diffusion method only; minimum inhibitory concentrations (MICs) are not reported.

**Table 4:** Summary: Anti-Inflammatory Therapeutic Area.

Compound	Substitution Pattern	Biological Model	Potency/Efficacy	Route of Administration	Observations/Comparison
(**13**–**15**)	Nitro group on ring A (isomers).	Carrageenan-induced plantar edema model in rats.	**14**: Maximum anti-inflammatory protective effect (MAPE) of 68.0% (at 1 h). **13**: Highest area under the curve (AUC: 378.08). **15**: MAPE ~60%.	Oral and intraperitoneal (i.p.). The i.p. route yielded superior outcomes.	**13**: Efficacy comparable to meloxicam with no statistically significant difference. **15**: Reduced efficacy compared to the reference drug.
(**16**–**17**) y (**6**)	Nitro group on ring B (isomers).	Carrageenan-induced plantar edema model in rats.	Dose-dependent protective effect (25 to 200 mg/kg). Maximal edema inhibition of ~70% at 200 mg/kg.	Oral and intraperitoneal (i.p.).	Effect of comparable magnitude to meloxicam (10 mg/kg, orally). Compound **17** exhibited the most pronounced anti-inflammatory effect.
(**13**), (**16**) y (**18**)	**13**: Nitro group in *ortho* position of ring A. **16**: Nitro group in *ortho* position of ring B. **18**: Nitro group in *ortho* position of ring A and *meta* position of ring B.	TPA-induced mouse ear edema (in vivo).	Inhibition of the inflammatory process: **13**: 71.17 ± 1.66%; **16**: 80.77 ± 2.82%; and **18**: 61.08 ± 2.06%	Topical (implicit in the TPA ear model).	Activity comparable to, or exceeding, the reference drug indomethacin (71.48 ± 1.62%). Statistically significant dose–response relationship.
(**14**)	Substituted on ring A.	Albumin denaturation inhibition model (in vitro).	Inhibition of approximately 89% at a concentration of 30 μg/mL.	In vitro.	Effect analogous to that of the reference drug.

**Table 5:** Summary: Analgesic Therapeutic Area.

Compound	Substitution Pattern	Target/Pathway	Biological Model	Potency/Efficacy	Selectivity/Observations	Key Limitations
(**19**)	3-nitro (Ring A)	Interaction with the μ-opioid receptor.	Acute pain models in mice (contortion/writhing test, hot plate test); SH-SY5Y neuroblastoma cell line.	Ki: 10.8 ± 3.6 μM for μ-opioid receptor. 96.1% inhibition of contortions at the highest dose (30 mg/kg).	Central and peripheral antinociceptive mechanisms. Not attributable to sedation or motor toxicity. No substantial cytotoxicity (74.8% viability at 20 μM).	Moderate affinity for the μ-opioid receptor (micromolar range).
(**20**) y (**21**)	4-nitro (Ring B)	Reduction in leukocyte migration, MPO activity, and inhibition of proinflammatory cytokines (IL-1β, TNF).	Murine models of acute and persistent pain (carrageenan, CFA, PSNL, and B16F10 melanoma); LPS-stimulated macrophages.	Significant inhibition of mechanical hypersensitivity, comparable to indomethacin. Compound 20 exhibited the greatest efficacy in the cancer model.	Selective mechanism: 20 curtailed hypersensitivity induced by IL-1β, TNF, epinephrine, and PGE_2_; 21 lacked efficacy against PGE_2_. No effect on IL-10 or KC/CXCL1.	None explicitly reported. Favorable pharmacokinetic profile (compliant with Lipinski and Veber’s rules).

**Table 6:** Summary: Antiparasitic Therapeutic Area.

Compound	Substitution Pattern	Target/Pathway	Biological Model	Potency/Efficacy	Observations	Key Limitations
(**22**) y (**23**)	4-nitro (Ring A)	Induces paralysis in nematodes.	Root-knot nematode (Meloidogyne incognita), second-stage juveniles.	EC_50_/24 h: 25 ± 17 mg/L for (**22**) and 71 ± 13 mg/L for (**23**).	The nitro group is identified as a pivotal component of the pharmacophore for nematicide activity.	Not explicitly reported.
(**13**)	2-nitro (Ring A)	Rapid lethality, inhibition of motility, mating (pair separation), and reproduction.	Adult Schistosoma mansoni (in vitro).	100% mortality at all evaluated concentrations (25–200 μg/mL) after 2 h.	A sublethal dose (25 μg/mL) induced 60% worm pair separation at 6 h and completely suppressed oviposition (0%).	Evaluated in vitro only; lack of in vivo validation in the described text.

**Table 7:** MIC of nitrochalcones **6** and **16** evaluated against different dermatophytes [41].

			MIC µg/mL		
Compounds	*Microsporum canis*	*Microsporum gypseum*	*Trichophyton mentagrophytes*	*Trichophyton rubrum*	*Epidermophyton floccosum*
**6**	0.75	1.5	1.5	0.75	0.25
**16**	12.5	6.25	6.25	12.5	3

**Table 8:** Summary Table: Antifungal Therapeutic Area.

Compound	Substitution Pattern	Target/Pathway	Biological Model	Potency/Efficacy	Observations/Selectivity	Key Limitations
(**6**) y (**16**)	Nitro Group *para* and *ortho* positions (Ring B)	Not explicitly reported.	Dermatophytes (e.g., *Epidermophyton floccosum*).	**6**: MIC = 0.25 µg/mL against *E. floccosum*.	Compound **6** surpasses the potency of the unsubstituted chalcone. Compound **16** remains superior to most non-nitro compounds.	Not explicitly reported.
(**24**–**26**)	Nitro Group *para* position (Ring B)	Not explicitly reported.	Fungi: *Aspergillus niger*, *Trichophyton rubrum*, and *Trichophyton mentagrophytes*.	**24**: MIC = 80 µg/mL (A. niger), 100 µg/mL (*T. rubrum*). **25**: MIC = 80 µg/mL (A. niger). **26**: MIC = 40 µg/mL (*T. mentagrophytes*).	Moderate antifungal activity combined with a favorable toxicity profile in cell lines.	Not explicitly reported.
(**27**–**30**)	Nitro group in *meta* and *para* positions of ring B and *ortho* positions of ring A.	Not explicitly reported.	Dermatophyte *Trichophyton rubrum* (main etiological agent of dermatophytosis).	**28** and **29**: MIC_80_ = 0.5 µg/mL. **27** and **30**: MIC_80_ = 16 µg/mL	Compounds **28** and **29** exhibit potent activity comparable to fluconazole (MIC_80_ = 0.25 µg/mL). Substituting position 2′ with amino groups yields superior outcomes than hydroxyl groups.	Not explicitly reported.

**Table 9:** Summary: Antileishmanial Therapeutic Area.

Compound	Substitution Pattern	Target/Pathway	Biological Model	Potency/Efficacy	Observations/Selectivity	Key Limitations
(**6**)	Nitro substituent in the para position of ring B.	Induces late apoptosis, increases ROS production, mitochondrial membrane depolarization, and phosphatidylserine exposure.	*Leishmania amazonensis* (promastigote forms).	IC_50_: 21.2 µM.	Significant morphological alterations (reduction in parasite cell volume).	Not explicitly reported.
(**6**) (Free and Solid Lipid Nanoparticles	4-nitrochalcone (para position of ring B).	Disruption of mitochondrial function, inhibition of macromolecule biosynthesis, and an increase in ROS in the parasite.	*L. infantum* and *L. amazonensis* (promastigotes); J774.1 macrophages.	Free compound significantly reduces promastigote viability. Nanoparticles act as a sustained-release system within macrophages.	Nanoparticles show no toxicity to J774.1 macrophages at <10 µM (reduced cellular toxicity compared to the free compound).	Nanoparticles lack a direct toxic effect on promastigotes (requires internalization by host macrophages).
(**31**) (Free and PLGA Microspheres)	3-nitro (Ring B).	Direct antiparasitic action (does not induce oxidative mechanisms like NO or ROS in host macrophages).	*L. amazonensis* (intracellular amastigotes); murine macrophages (in vitro).	IC_50_: ~10 µg/mL against intracellular amastigotes.	Microspheres (<6 µm) are efficiently internalized by macrophages within 3 h. Encapsulation significantly reduces toxicity to macrophages, improving the safety profile.	Not explicitly reported.

**Table 10:** Cytotoxicity of **34–36** against cell lines A549, MCF-7, and HEK293-T [50].

		IC_50_ Values (μM ± SD)		
Compounds	A549	MCF-7	HEK293-T	VEGFR-2
**34**	52.16 ± 2.40	23.33 ± 0.15	22.61 ± 0.17	3.59 ± 0.02
**35**	17.55 ± 0.08	11.98 ± 0.09	32.61 ± 0.14	3.76 ± 0.01
**36**	34.52 ± 0.42	12.08 ± 0.54	36.31 ± 0.15	5.36 ± 0.02
Estaturosporin	0.22 ± 0.02	0.25 ± 0.02	0.87 ± 0.04	5.18 ± 0.003
Nintedanib	0.46 ± 0.06	0.44 ± 0.03	0.98 ± 0.08	6.87 ± 0.002

**Table 11:** Summary: Cytotoxic Therapeutic Area.

Compound	Substitution Pattern	Target/Pathway	Biological Model	Potency (IC_50_)	Observations/Selectivity	Key Limitations
(**32**)	dinitro in *para* positions on both rings	Inhibition of cell proliferation	Human nasopharyngeal carcinoma cell line (KB)	12.3 µM	Surpasses the positive control 5-fluorouracil (IC_50_ = 16.9 µM).	Not explicitly reported.
(**33**)	*Ortho* position (Ring B)	Inhibition of cell proliferation	Rhabdomyosarcoma cell line	7.86 µM	Comparable anticancer potency to doxorubicin and methotrexate (no statistically significant difference, *p* > 0.05).	Not explicitly reported.
(**6**)	4-nitro (Ring B).	Enhanced internalization mediated by folate receptors	HeLa cells; NIH3T3 fibroblasts; human erythrocytes	Free: 46.7 µM. Encapsulated: reduces viability by ~30% and 63% at 15 and 30 µM	Selective profile: no significant toxicity in non-tumor NIH3T3 cells or human erythrocytes up to 50 µM. Encapsulation significantly enhanced the effect.	Free compound shows only moderate dose-dependent toxicity.
(**34**–**36**)	*meta* position (Ring A).	Potential to inhibit VEGFR-2 tyrosine kinase.	Lung (A549) and breast (MCF-7) cancer cells; HEK293-T (non-cancerous)	34: 13.36 ± 0.29 µM (A549). 35: 11.98 ± 0.09 µM (MCF-7). 36: 12.08 ± 0.54 µM (MCF-7).	Significantly reduced toxicity in non-cancerous HEK293-T cells. Moderate toxicity compared to reference drugs.	Not explicitly reported.

**Table 12:** Influence of the nitro group on the positions of rings A and B in nitrochalcones (hypothesis-generating).

Ring	Position of NO_2_	Electronic Effect	Steric Effect	Impact on Biological Activity	SAR Conclusion
Ring B	*para*	Optimal conjugation with the α,β-unsaturated system	Minimum	High multitarget potency (anticancer [25,26,27,28], anti-inflammatory [34], vasorelaxant [4]), antimicrobial [30], analgesic [38], antifungal [43], cytotoxic [47].	Most favorable position; privileged core
Ring B	*meta*	Limited conjugation	Minimum	Moderate or selective activity [4,34,43,46]	Functional position but less powerful
Ring B	*ortho*	Conjugation affected by torsion	High	Variable activity; possible selectivity [4,29,30,34,50].	Ambivalent effect
Ring A	*para*	Indirect electronic modulation	Minimum	Increased selectivity rather than potency [31,32,33,39,47].	Modulating role
Ring A	*meta*	Limited electronic effect	Minimum	Moderate activity [33,36,50].	Side effect
Ring A	*ortho*	Conformational alteration	High	Selective or reduced activity [4,33].	Conformational influence

## Data Availability

The data presented in the study are available in the article.
